# SWPepNovo: An Efficient De Novo Peptide Sequencing Tool for Large-scale MS/MS Spectra Analysis

**DOI:** 10.7150/ijbs.32142

**Published:** 2019-07-03

**Authors:** Chuang Li, Kenli Li, Keqin Li, Xianghui Xie, Feng Lin

**Affiliations:** 1 College of Information Science and Engineering, Hunan University, Changsha, China;; 2 College of Information Science and Engineering, Hunan University, National Supercomputing Center in Changsha, Changsha, China.; 3 College of Information Science and Engineering, Hunan University, Department of Computer Science, State University of New York, NY, USA.; 4 State Key Laboratory of Mathematic Engineering and Advance Computing, Wuxi Jiangnan Institute of Computing Technology, Jiangsu, China.; 5 School of Computer Science and Engineering, Nanyang Technological University, Singapore.

**Keywords:** Large-scale MS/MS spectra analysis, de novo peptide sequencing, high performance computing, SW26010

## Abstract

Tandem mass spectrometry (MS/MS)-based de novo peptide sequencing is a powerful method for high-throughput protein analysis. However, the explosively increasing size of MS/MS spectra dataset inevitably and exponentially raises the computational demand of existing de novo peptide sequencing methods, which is an issue urgently to be solved in computational biology. This paper introduces an efficient tool based on SW26010 many-core processor, namely SWPepNovo, to process the large-scale peptide MS/MS spectra using a parallel peptide spectrum matches (PSMs) algorithm. Our design employs a two-level parallelization mechanism: (1) the task-level parallelism between MPEs using MPI based on a data transformation method and a dynamic feedback task scheduling algorithm, (2) the thread-level parallelism across CPEs using asynchronous task transfer and multithreading. Moreover, three optimization strategies, including vectorization, double buffering and memory access optimizations, have been employed to overcome both the compute-bound and the memory-bound bottlenecks in the parallel PSMs algorithm. The results of experiments conducted on multiple spectra datasets demonstrate the performance of SWPepNovo against three state-of-the-art tools for peptide sequencing, including PepNovo+, PEAKS and DeepNovo-DIA. The SWPepNovo also shows high scalability in experiments on extremely large datasets sized up to 11.22 GB. The software and the parameter settings are available at https://github.com/ChuangLi99/SWPepNovo.

## Introduction

In post-genomic era, proteomics has become the most active research fields, and mass spectrometry has developed into a leading technology for large-scale analysis of proteins, including high-throughput analysis of proteins and determination of their primary structures^1^. There are two basically methods for protein analysis using MS/MS spectra: database-search based peptide sequencing and de novo peptide sequencing 2.

Database-search based peptide sequencing, which aims at retrieving all candidate sequences from a specified protein sequence database for each MS/MS spectrum 3, is a widely used method for protein analysis [4][5][3][6]. In database searching, it is generally assumed that the genomes are precisely sequenced, and the protein-coding genes and RNA genes are just annotated completely. But the latter is not satisfactory because a lot of alternatively spliced genes do not exist in now available databases 7. The major limitation of this method is its highly dependence on the protein database. In addition, due to the use of the relatively simple scoring modules, it is easy to miss the identification by database searching. Thus, the database searching methods cannot provide a complete solution for protein analysis.

Many efforts have directed their attention to the development of de novo sequencing methods for protein analysis. Various de novo sequencing methods such as PepNovo+ 7, PEAKS 8, pNovo 9, and UniNovo 10 have been developed in recent years. De novo sequencing can directly extract a protein sequence from a MS/MS spectrum without knowledge of the organism or even the genomic sequences^11^, and it can process post-translational modifications (PTMs), sequence variations and the mass spectra with low signal-to-noise ratio that cannot be effectively processed by database searching methods^12^. As a consequence, de novo peptide sequencing, as an irreplaceable tool to discover new proteins and PTMs, has been widely acknowledged in the research of proteomics at present.

However, the number of MS/MS spectra data has been increasing sharply benefits from the technological breakthroughs of the modern spectrometry in recent years 13. Besides, the protein and peptide analysis criteria have become more demanding, e.g. with chemical and post translational modifications and/or when considering enzyme semi-unconstrained searches^14^. Accordingly, analyzing this huge amount of MS/MS data using de novo peptide sequencing becomes a significant challenge for proteome researchers. Without developing more powerful and efficient de novo peptide sequencing algorithms, the computational bottlenecks that we can expect is that the scope of discoveries will be limited to small-scale MS/MS spectra data. Breakthrough of efficient de novo sequencing method is crucial for large-scale protein analysis in computational biology 15.

Fortunately, various high performance computing systems (HPCS) such as Intel Many Integrated Core Architecture (MIC) 16 and Graphics Processing Unit (GPU) 17 have recently been developed to improve the computational efficiency. GPU can support parallel programming language such as Open Computing Language (OpenCL) 18 and Compute Unified Device Architecture (CUDA)^19^, which is widely-used in computational biology research. MIC architecture, which contains 60+ cores and 512-bit-wide vector units, is a coprocessor designed to highly parallel multithreaded application with high memory requirements. A similar architecture, SW26010 many-core processor, has recently been developed at National Research Center of Parallel Computer Engineering & Technology for protein and peptide analysis. This paper proposes an efficient parallel PSMs algorithm for large-scale MS/MS spectra data analysis on SW26010. The main contribution and innovation of this study can be summarized as follows:

- We design and implement the parallel PSMs algorithm using a two-level parallelization mechanism. To our best knowledge, our algorithm is the first attempt to improve the efficiency of large-scale MS/MS spectra data analysis and processing.
- We present a high-effective structural optimized MS/MS data organization to overcome the memory access bandwidth bottleneck and propose a highly scalable intra-MPE communication scheme, which gets a parallelization efficiency of over 85%.
- We adopt the SW26010 processor for large-scale protein analysis that uses the parallel PSMs algorithm. In design realization, we also employ asynchronous task transfer and propose a series of effective optimization strategies to decrease the communication costs between the management processing elements (MPEs) and computing processing elements (CPEs) and to balance the workload on each CPE, which results in a 10× speedup compared with the un-optimized version.
- We also prove the scalability of SWPepNovo by scaling the size of datasets and the number of SW26010 nodes. We obtain an ideal speedup on a multi-node cluster that contains three SW26010 processors with a total of 4096 CPE. Experimental results show that our method has an excellent performance on scalability and without sacrificing accuracy and correctness in the de novo peptide sequencing results.

We believe that the techniques we use can guide the design of similar work on the neo-heterogeneous SW26010 many-core architecture. The software and the parameter settings are available from *https://github.com/ChuangLi99/SWPepNovo*. Users without access to TaihuLight, SWPepNovo can be run as a multi-threaded (OpenMP) application on a MPI cluster.

The rest of this paper is organized as follows. Section II gives the MS/MS-based de novo peptide sequencing, the Sunway TaihuLight supercomputer and the related work. Section III provides details of computational design and optimization strategies. The experiment performance is evaluated in Section IV. Finally Section V, we validate our results with a previous study and conclude the paper.

## Backgrounds

In this section, we started with an introduction to the background knowledge for MS/MS-based de novo peptide sequencing and SW26010 many-core processor (The main processor of the Sunway TaihuLight Supercomputer), and then present the existing parallel works in protein and peptide mass spectra analysis.

### De novo peptide sequencing

**Figure 1:**
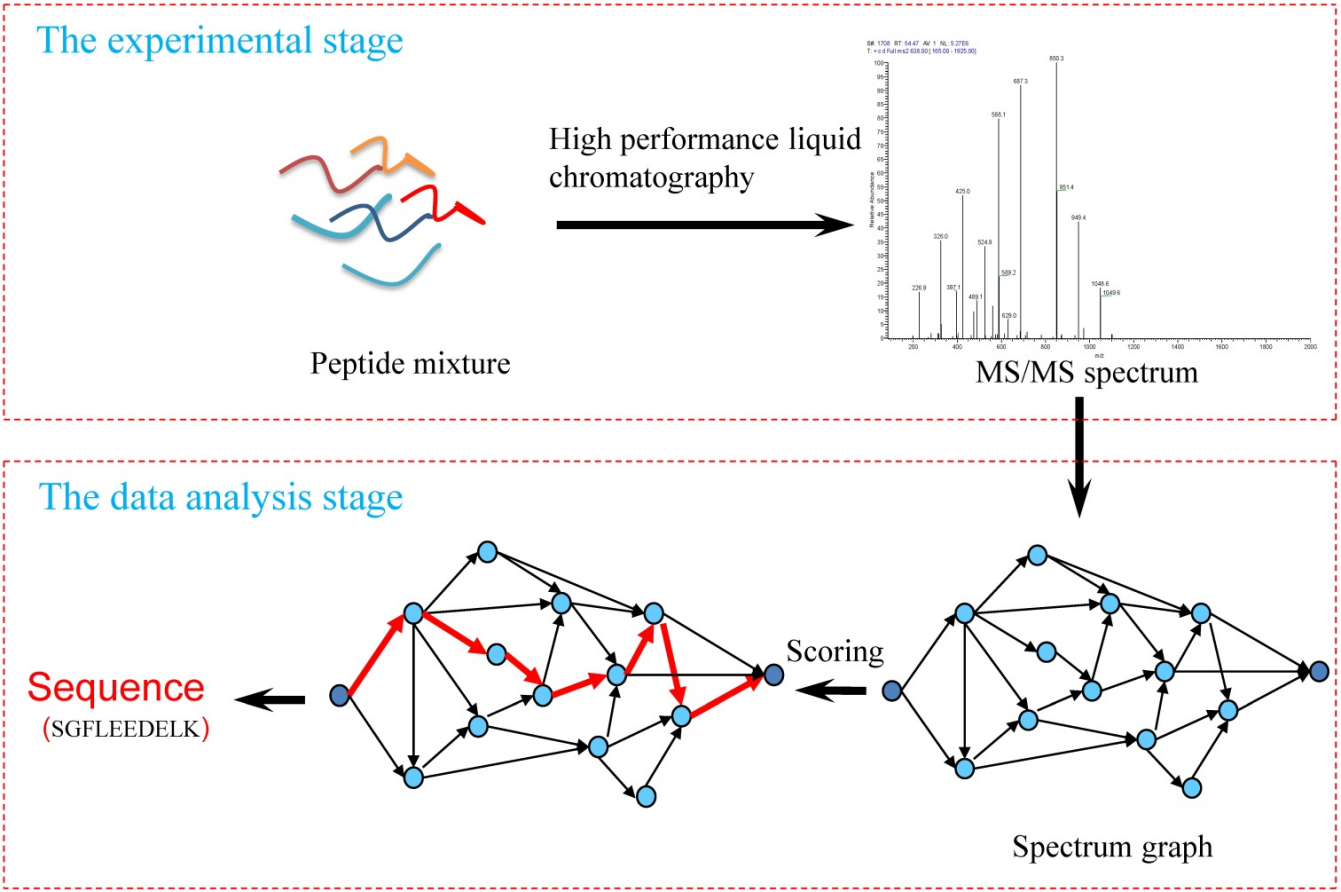
Workflow of the de novo peptide sequencing.

**Figure 2:**
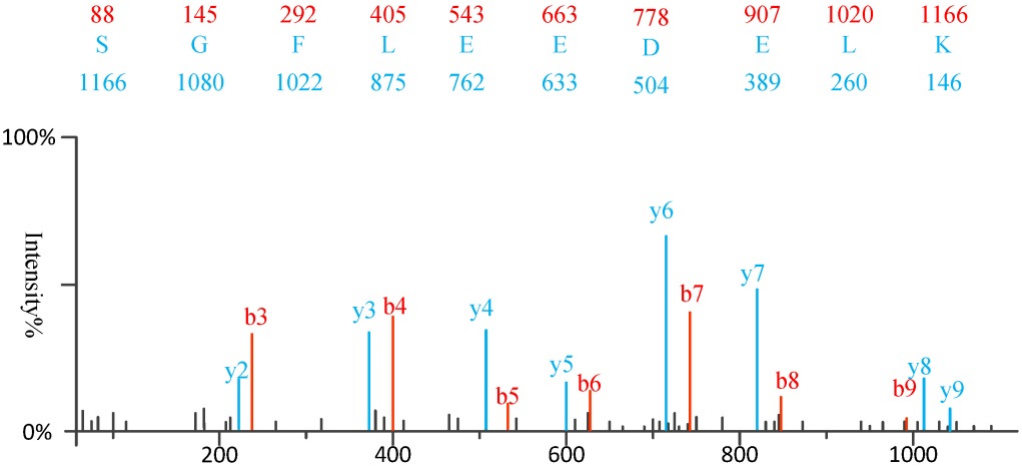
An example of MS\MS spectrum

**Figure 3:**
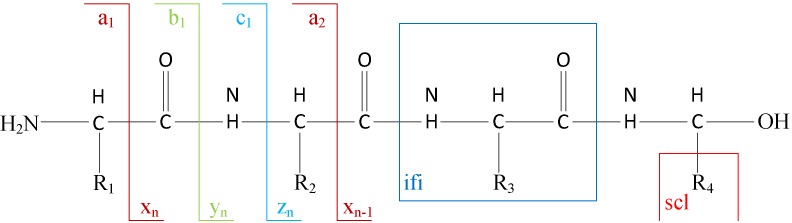
The type of fragmentation ions.

De novo peptide sequencing aims to deduce an amino acid sequence according to MS/MS spectrum without the use of a protein sequence database. Figure 1 shows the processing flow of MS/MS spectra analysis using de novo sequencing methods, which mainly includes three key parts:

1) Experimental spectra generation: First, the mixed proteins digest into mixed peptides using by enzymes. And then the peptides will be fragmented and ionized (e.g., higher energy collisional dissociation (HCD) 20, collision-induced dissociation (CID) 21) in liquid chromatography tandem mass spectrometry (LC-MS/MS). Finally, the MS/MS spectra will be output. Figure 2 is a MS/MS spectrum, which contains the measured m/z and intensity of the fragments, represented by the peaks 10. Different ionization methods have dramatic impact on the propensities for producing particular fragment ion types. For example, in CID, there are six series of fragment ions, which are denoted by type fragments C-terminal x, y and z and N-terminal a, b and c type fragments 12, as shown as Figure 3.

2) Spectrum graph generation: The spectrum graph is constructed through transforming an effective peak set into a spectrum graph where each node in spectrum graph is the m/z value. The nodes are connected if the difference of the two m/z values equals to an amino acid mass.

3) Match scoring: First, the candidate peptides are reconstructed based on the spectrum graph. A candidate peptide is a path where the accumulative value of the weights equals to the parent mass. Then, the similarity of the experimental MS/MS spectrum and candidates are calculating using scoring algorithm, the top one(s) is the result of the calculation. Finally, the identified MS/MS spectrum was merged as the final peptide sequence.

### Sunway TaihuLight supercomputer

The Sunway TaihuLight supercomputer, which developed by the NRCPC, is now ranked third in the latest TOP 500 list of October 2018, and have installed in the National Supercomputing Center in Wuxi. The peak performance of Sunway TaihuLight is 125.436 Pflops. The sustained LINPACK performance is 93.015 Pflops, leading to a performance-per-watt of 6051 MFLOPS/W 22. The Sunway TaihuLight architecture consists of four levels: the entire computing system, a super node, a cabinet, and a computing node. All computing nodes in entire computing system are connected with Sunway Network which is a customized network. A total of 1024 TB of memory and 20PB of storage are in Sunway TaihuLight. The system software is a 64-bit Sunway RaiseOS 23.

**Figure 4:**
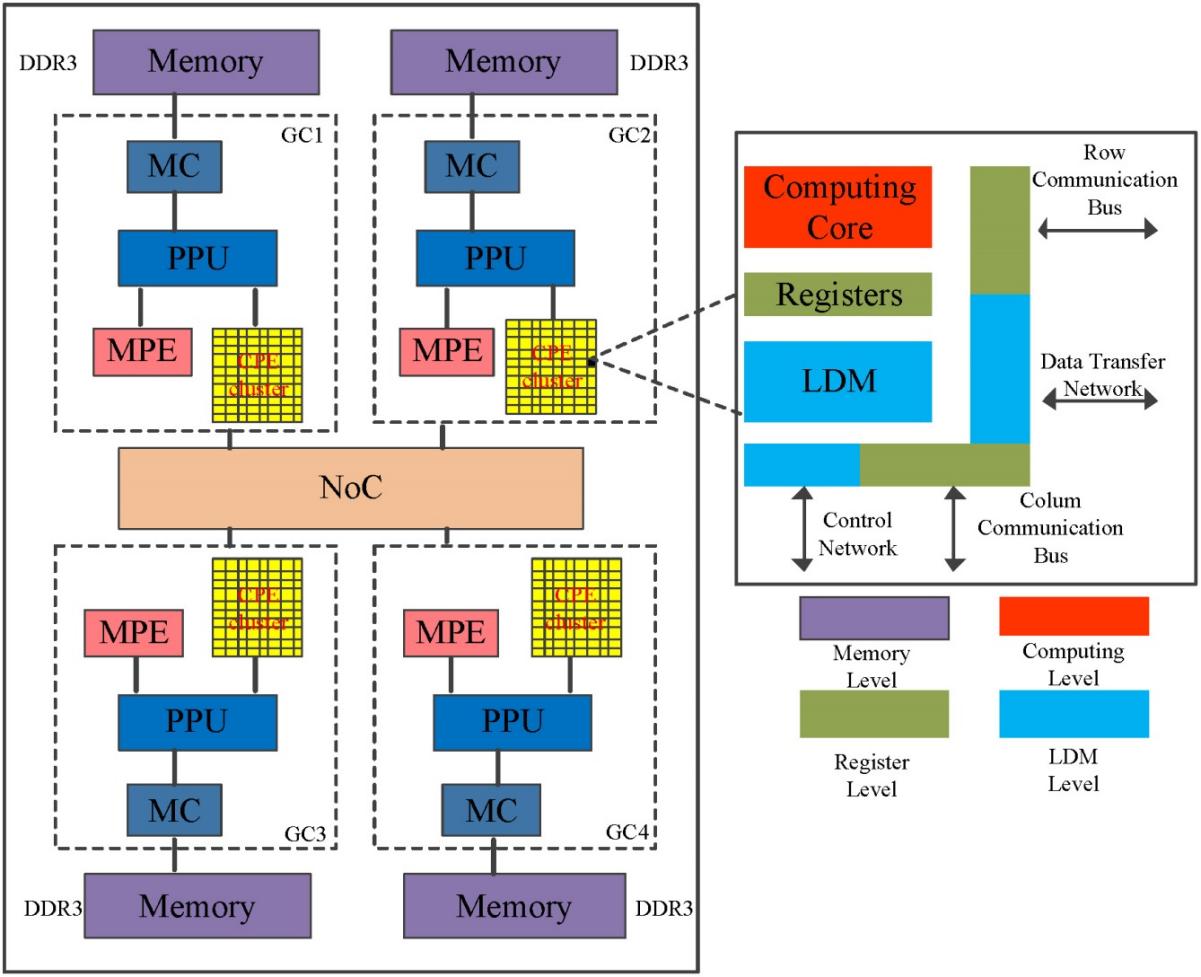
The architecture of the SW26010 manycore processor.

The SW26010 is the core of an embedded Sunway TaihuLight system^24^, which the general architecture is illustrated in Figure 4. The processor includes four core-groups (CG) which equip with a single management processing element (MPE) and 8x8 computing processing elements (CPE), one memory controller (MC) and 8 GB physical memory 22. SW26010 processor supports two user programming modes: (1) Chip-sharing mode; and (2) CG private mode. The Chip-sharing mode offers specific for applications with high memory requirements. In its CG private mode, the SW26010 processor serves as a NUMA (Non-uniform memory access) Architecture.

To some degree, the programming on CPE cluster is similar to GPU. MPE plays the role of the normal CPU and is mainly responsible for task management, Input/Output and communication. CPE cluster play the role of the accelerate card which determine the computational power 25. The peptide spectrum match scoring in de novo peptide sequencing requires more computing power. To run de novo peptide sequencing with an favorable performance, the CPE's optimization mechanisms are indispensable.

### Related Works

Previous research has shown that many efforts of the acceleration of protein identification are focused on developed parallel database searching based peptide sequencing. Lee adopted the graph-based in-memory distributed system to develop a novel sequence alignment algorithm 26. Qi You developed a fast tool that can highly efficient analyze genome-editing dataset 27. In 28, Li considered the redundant candidate peptides in PSMs, and adopted inverted index strategy for speeding up tandem mass spectrometry. And also, some of the prevalent peptide sequencing methods adopted high performance computing (HPC) technology and cloud computing 29. Notably, Zhu presented an efficient OpenGL-based Multiple peptide sequence alignment implementation on GPUs hardware 30. In^31^, a based-GPU feature detection algorithm was presented by Hussong to reduce the running time of PSMs.

As another most powerful method for protein analysis, de novo peptide sequencing has drawn limited attention in proteomics. A pioneering research on speeding up de novo peptide sequencing was done by Frank 32. In 32, Frank presented a discriminative boost ranking-based match scoring algorithm, which using machine learning ranking algorithms and producing identical speedup results while maintaining the same identification result. Another efficient real-time de novo sequencing algorithm, namely Novor, was recently presented by Ma 33. Compared with other de novo peptide sequencing methods, Novor shows a very fast sequencing speed. PEAKS 8, which developed by Ma, does the best job as acceleration de novo peptide sequencing. Although Peaks achieves great performance both in the speed and accuracy of de novo peptide sequencing analyses, it is a commercial software which cannot freely available to academic users.

Recently Sunway TaihuLight supercomputer provide tremendous compute power to researchers. There are a few early development experiences on Sunway TaihuLight supercomputer. Chen et al. 34 designing and implementing a parallel AES algorithm, and the result shows that the parallel AES algorithm achieved a good speed-up performance. Fang et al. 35 have implemented and optimized a library, namely swDNN, which that supports efficient deep neural networks (DNNs) implementation on Sunway TaihuLight supercomputer. In 36, a SW26010-based programming framework was presented for Sea Ice Model (SIM) algorithm. According to the experiment results, the programming framework for SIM algorithm offers up to 40% performance increase.

## Computational Design

**Figure 5:**
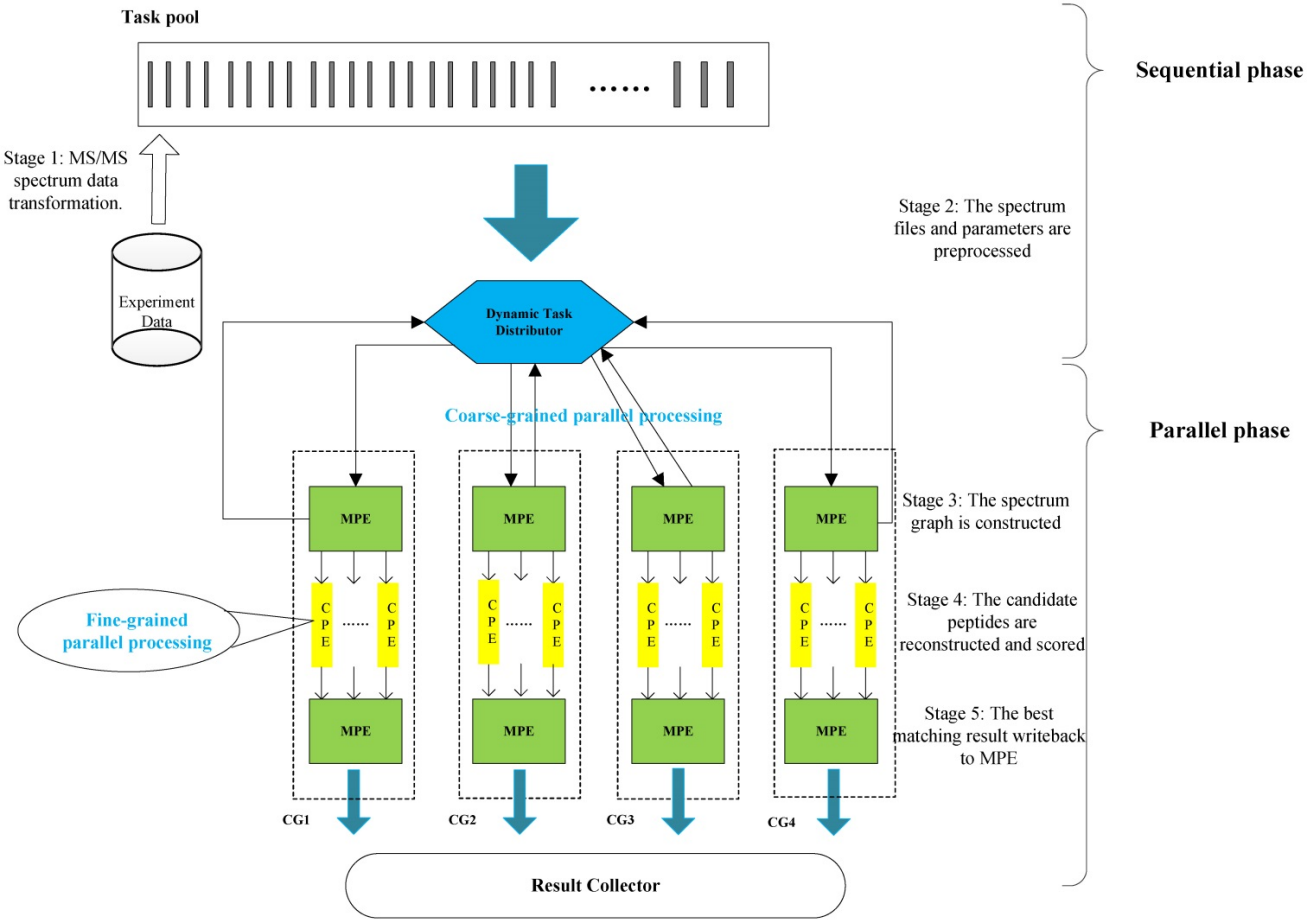
The algorithm framework of our implementation.

In this section, we present the efficient de novo peptide sequencing for large-scale MS/MS spectra analysis on SW26010. Our algorithm design is benefits of both the fact that the inherent parallelism of the match scoring progress and each CPE in CG enables eight single-precision or simultaneous double-precision floating points operations, and combines: (1) the task-level parallelism between MPEs using a data transformation method and a dynamic feedback task scheduling algorithm, (2) thread-level parallelism across CPEs using asynchronous task transfer and multithreading. The diagram in Figure 5 illustrates the algorithm framework of our implementation.

Our two-level parallelization scheme on SW26010 many-core architecture combines: (1) task-level parallelism between MPEs using a dynamic feedback task distribution method (based on MPI), and (2) thread-level parallelism across CPEs (based on aThread).

### The Task-level Parallelism between MPEs

In the task level parallel part, the dataset is divided into properly-sized chunks depending on the length of peptide and the integral multiple of CG when handling a large-scale MS/MS spectra dataset. Then, all MPE in CG continues to process these chunks in a coarse-grained parallel fashion. Since each CG in SW26010 has its own dedicated random access memory (RAM), and the communication mode is limited to register communication, assignment and scheduling of tasks is critical to improve the parallel performance. To support de novo peptide sequencing tasks for large-scale dataset effectively and efficiently, we use a MS/MS data transformation method to optimize the original sequences reading format and implement a dynamic distribution framework to assign MS/MS data chunks to MPE. The precise details about data transformation and dynamic distribution framework are in subsections later.

#### Data Transformation

**Figure 6:**
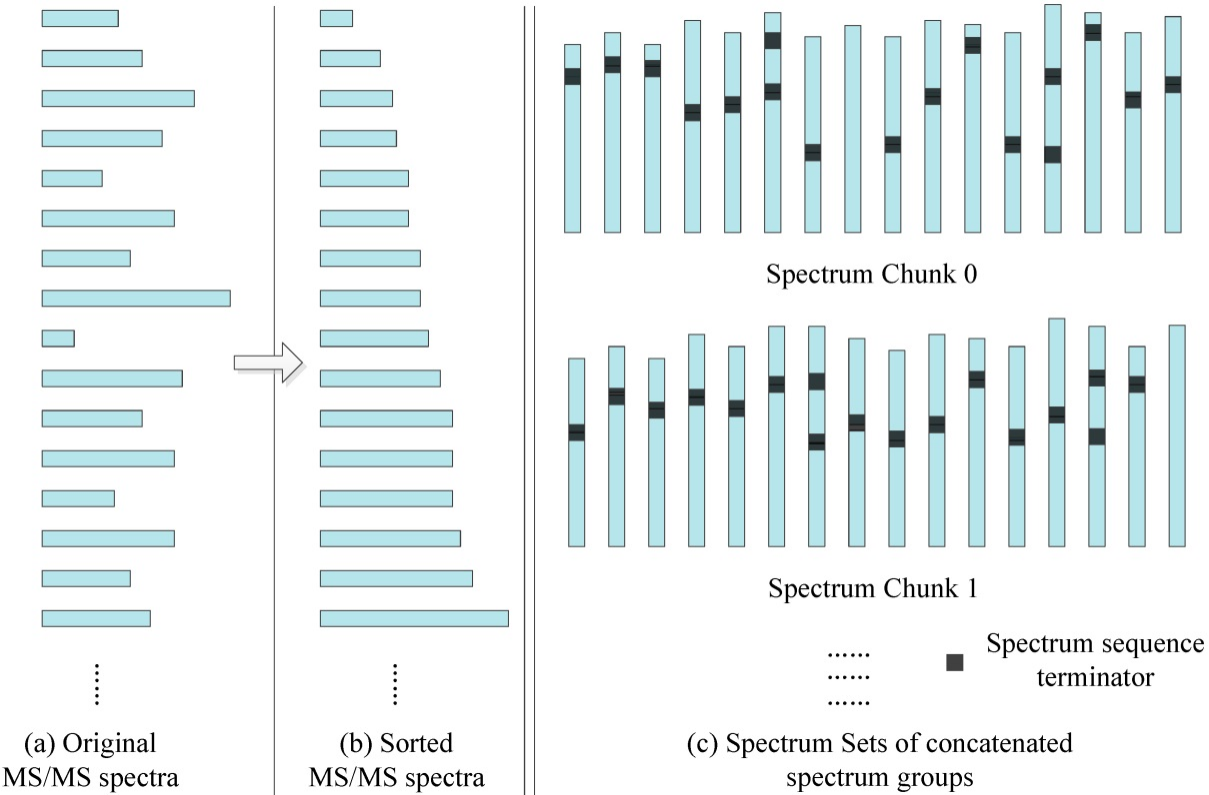
The MS/MS data transformation.

In order to better match the SW26010 capabilities, our parallel implementation is not directly loading MS/MS spectra dataset, but transforming them into a format. As the Figure 6 shows, the transformation process consists of two steps: Sorting by spectrum sequence length, and concatenation.

**Sorting:** In the SW26010 processor architecture, each MPE manages 64 CPE for parallel processing. This means that the threads in CPE will have to wait for each other to finish their workload instead of continuing on independently. As shown, for the purpose of shortening the waiting time, all the MS/MS spectrum are sorted by the length of spectrum to minimize deviation between neighboring threads. The time complexity is *O(N^2^)*, where *N* is the quantity of the amino acid sequences of this sorting process.

**Concatenation:** Even though sorting by length has somewhat balanced workload in each MS/MS spectrum, various MS/MS spectrum still have different parent mass. To overcome this issue, spectrum within a spectrum groups are concatenated with spectrum to form spectrum groups. The lengths of spectrum groups within a spectra chunk are nearly equal to the size of the biggest spectrum in that set. Each thread will achieve workload balancing by inserting spectrum terminators between the concatenated spectra. The time complexity is *O(N^3^)*, where *N* is the quantity of the amino acid sequences of this sorting process.

#### Dynamic Distribution Framework

**Figure 7:**
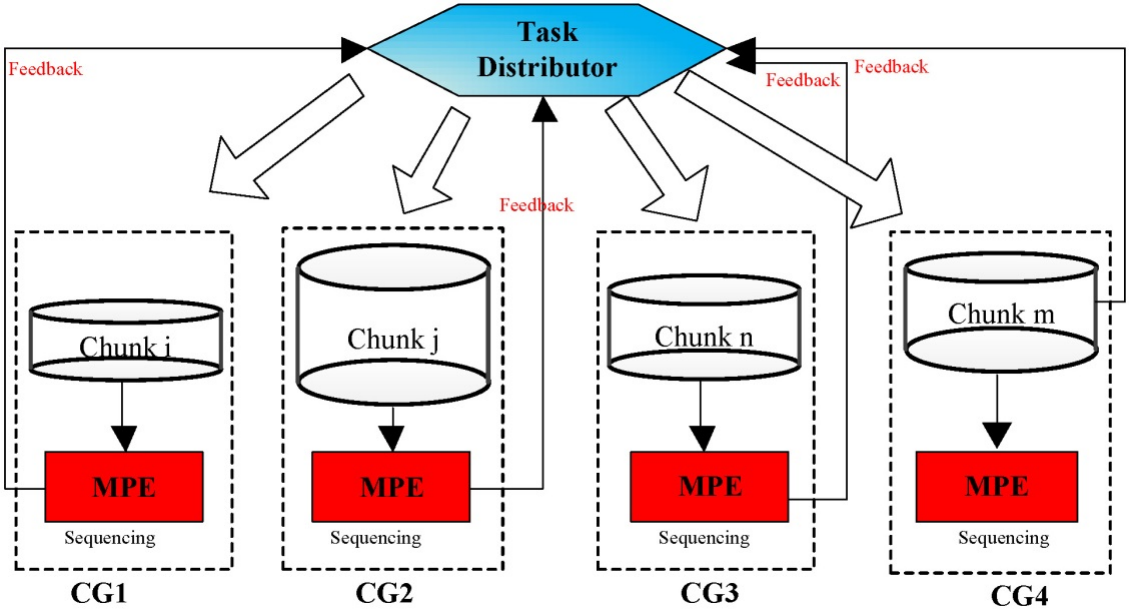
A flowchart of the task distribution framework.

As is shown in Figure 7, a random set of chunks is executed to explore the availability of CGs, which includes information about computing time and load balancing. Then, based on feedback in the previous step, we have defined a feedback regulatory factor for each CG. Finally, we adjust the amount of the chunks assigned to the CG according to the feedback regulatory factor of each MPE.

**Figure 8:**
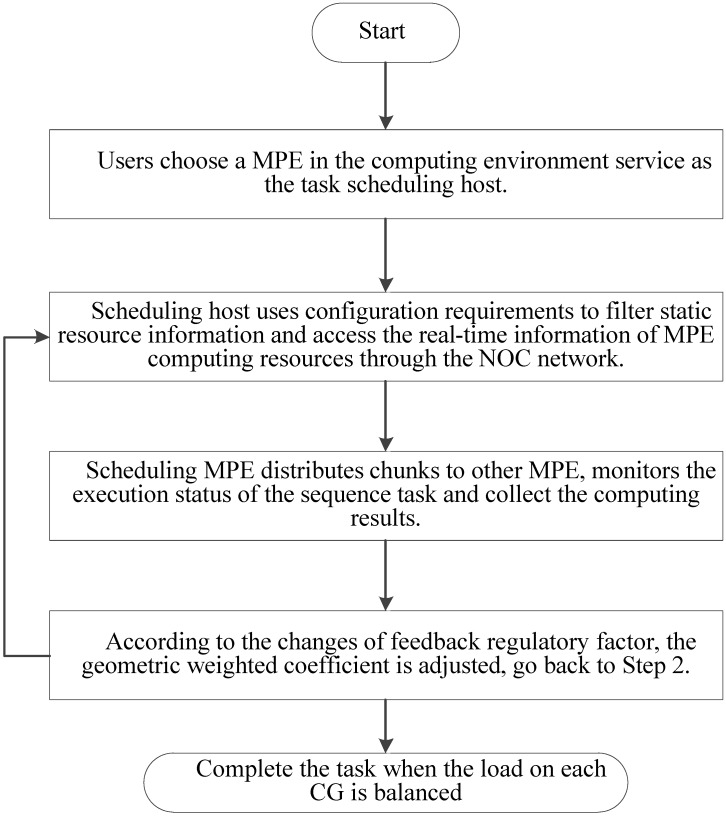
Feedback and adjustment mechanism based task dynamic scheduling process.

In implementation, choosing an appropriate load parameters as the feedback regulatory factor is very important to eliminate system bottleneck and balance the load dynamically. Our priority is the CPE of SW26010 processor utilization. In order to calculate CPE utilization, we have extracted the real-time information parameters from the file */proc/stat/*. Besides, since the length of task queue decides that whether the scheduler can keep the consistence with the system requirements. Too long computing time of a task queue can result that the many-core system will be in the state of overload. Thus, the average task queue length is also important to the feedback regulatory factor. In our experiment, we obtained the single CPE average queue length from the related parameters in file */proc/loadavg/*. The detailed steps of dynamic feedback task scheduling process are described in the Figure 8. The experimental results show that the dynamic feedback task distribution can keep the system load imbalance below 9 percent in most cases.

#### The intra-MPE Communication Scheme

**Figure 9:**
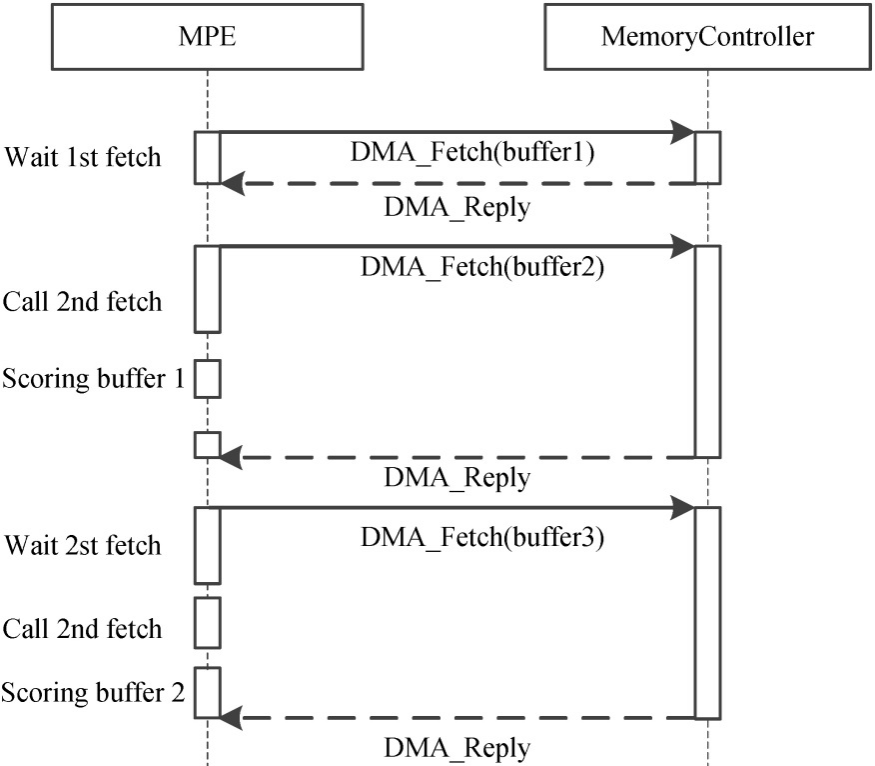
Asynchronous data transfer strategy. When an chunk in one buffer is scored, the subsequent chunk is being fetched to the other buffer using DMA-fetching intrinsics.

Because of the CPEs do not have cache and the latency of accessing DDR3 memory is fairly high, how to explicitly manage the use of local device memory (LDM) in the SW26010 architecture is critical. Direct memory access (DMA) has been widely acknowledged as an efficient method to transfer data between LDM and memory. Thus, we adopt the asynchronous DMA-fetching strategy, which presented by Sunway TaihuLight, to overlap data transfers from shared memory to local device memory. Figure 9 illustrates this strategy.

### The Thread-level Parallelism across CPEs

**Figure 10:**
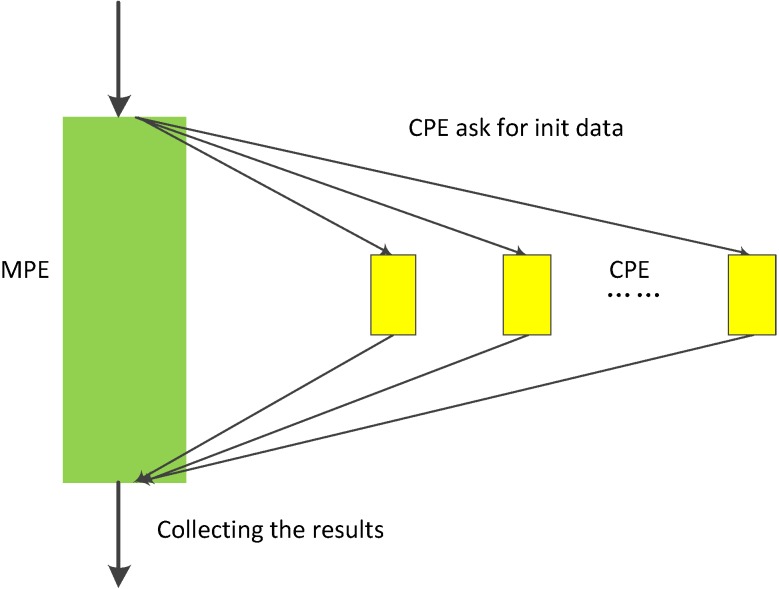
The dynamic parallel programming model

In neo-heterogeneous SW26010 many-core architecture, the CPE cluster plays the role as a coprocessor which dominates the computing power. In order to fully utilizing the parallel super-computing power of the CPE cluster, it is vital to coordinate the relationship between CPE and MPE. SW26010 supports four kinds of programming models to combine CPE and MPE, which can be used to implement and optimize parallel application. By employing the dynamic parallel mode, the task distribution between the CPEs should be taken seriously. In our implementation, we adopt the dynamic parallel programming model, which is shown in Figure 10. MPE and CPE in SW26010 serve different functions during the computation. MPE is serving as a job manager, which responsible for tasks allocations, and CPE used as computing node is primarily responsible for receiving and executing tasks and returning results to the corresponding MPE.

In the thread level parallel part, the chunks are consecutively loaded into a separate CG for de novo sequencing. The peptide sequencing executed on the CG consists of four phases. In the first phase, each MS/MS spectrum obtains the spectrum graph in MPE. The spectrum graph is constructed through transforming an effective peak set into a spectrum graph where each node in spectrum graph is the *m/z* value. The nodes are connected if the difference of the two *m/z* values equals to an amino acid mass. In the second phase, the candidate peptide dataset are reconstructed based on the spectrum graph. A candidate peptide is a path where the sum of the weights equals to the parent mass. In the third phase, each MPE distributes each spectrum graph and the corresponding candidate peptides to the CPE cluster. In the last phase, the candidate peptides are scored in CPE cluster. The pseudo-code of the parallel PSMs algorithm shown in table A the algorithm 1. The parallelization at this level is implemented through a special accelerate thread library, called aThread.

**Figure 11:**
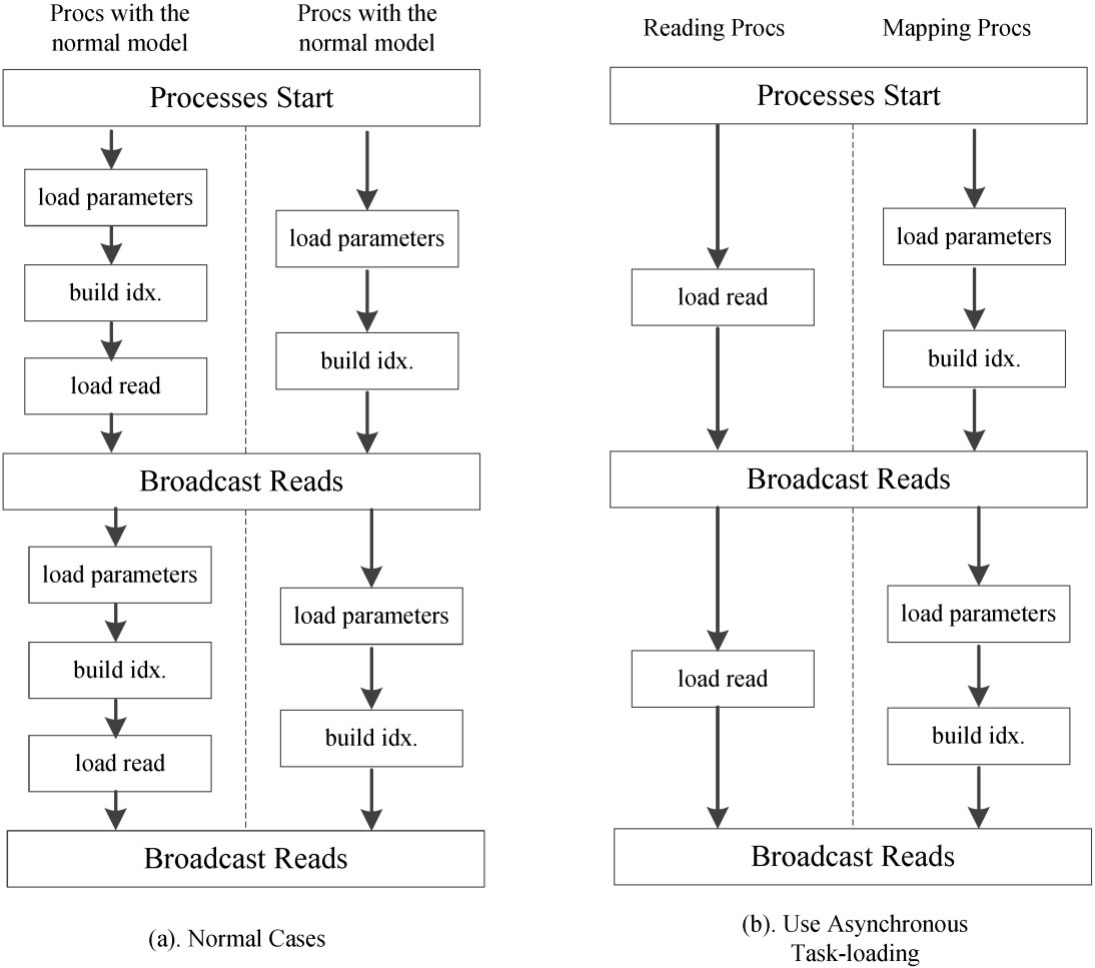
Asynchronous task-loading strategy.

We further employ an optional asynchronous task-loading strategy as shown Figure 11. First we set a list of processes to load only reads. When other processes on the active run-queue are computing scores, these processes is responsible for loading new read spectra chunks. Once the loading and computation are completed, all processes in the run-queue will receive the load data. When using 8 processes in our experiments, this strategy reduces the computational idle time between two read blocks by more than 85%.

## Optimization Strategies

In our experiments, the dataset size up to 10 GB and the excessive data replication exist in scoring process. Thus, a native parallel implementation is not a perfect solution. To get better performance for large-scale de novo peptide sequencing, we employ three optimization strategies, including vectorization, memory access optimization, and double buffering. The performance increase of optimization strategies is shown in Table I.

### Vectorization

Vectorization is critical to run the codes efficiently in neo-heterogeneous systems. In the Sunway TaihuLight architecture, each CPE in CG can process eight floating point operations within an instruction cycle. SW26010 many-core processor is specially offered SIMD processing unit and corresponding instructions. Moreover, the original automatic vectorization does not support an efficient binary file generation. Therefore, vectorization is a key point of the optimization process for efficient implementation of de novo peptide sequencing.

In our implementation, since the data dependence exists in the innermost loop, the first task is modifying dependent statements to eliminate data dependence. Then, too achieve an efficient utilization of all available CPEs computing resources by utilize vectorization, we adopt inter loop vectorization operation to manually expand the inner loop. When the variable mapping operations has occurred in function, SIMD store or SIMD load, the standard type must be a 32 bytes boundary alignment. In the practice implementation code process, we derive a data padding to make each memory access to be naturally aligned natural. As the key of the entire optimization process, the vectorization technique achieved a performance of 57 Glops.

### Memory Access Optimization

As described in Figure 4, each CPE contains a user controlled Scratch Pad Memory. The improvement of replacing caches by Controlled Scratch Pad Memory is more initiative and efficient for programmers. Meanwhile, the compiling system supports a collective communication interface and DMA intrinsic, which provides asynchronous transmission mode between the Scratch Pad Memory in CPE and main memory of MPE. If the sum of data transmitted is equal to the multiple of 128 byte, the limit on maximum peak performance of DMA intrinsic can achieve. Typically DMA supports three kinds of models to transmit data. In its single-CPE mode, each Scratch Pad Memory exchanges data within main memory individually. In its broadcast mode, data in the main memory are scheduled to CPEs. The single-row mode, each row of SPMs transfers data with main memory. In our work, we have used the single-CPE mode to design and implement parallel PSMs algorithms which can make full use of the compute resource.

During the optimization process, each Scratch Pad Memory sequentially receives a dataset of candidate peptides from main memory. Each CPE completes scoring and sends the top one to main memory respectively. Note that the number of data transferred must be a multiple of 128 byte. The DMA intrinsic can reduce the number of memory access during each round of scoring. We have obtain optimal performance and get higher speedup of SW26010 using DMA intrinsic.

### The Double-buffering Mechanism

Although the DMA intrinsic can be reduce the cost of memory access, the parallel efficiency and scalability still have a huge margin of improvement, especially in the part of candidate peptides scoring. For best performance and minimal communication consumption, we have used the double-buffering mechanism to overlap communication cost and computation cost. Our design is based on the following insights.

1. In Scratch Pad Memory, CPEs will allocate a double memory space to accommodate the data of 2 groups when the multi-cycle DMA has read/write operations.

2. The typical approach to hide memory access performance is to make the two sets buffer from each other. When one set serves as the message buffer, the other one is calculated.

3. The DMA bandwidth between LDM and memory are affected by initial dimension of loops. And a memory space in Scratch Pad Memory is required for data buffered storage when several rounds of DMA has a high number of write and read operations.

Based on the above design philosophy, we have implemented the double-buffering mechanism in parallel PSMs algorithm to accelerate de novo peptide sequencing. In our implementation, the memory access overhead in double buffering mechanism divided into two parts: unsheltered part *P*, which includes all the cost of transmitting the data in the first round and last round. Another is the overlapping cost part P * (N - 1). Eq. (1) shows the speedup of optimization by using the double buffering mechanism:


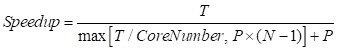
(1)

In the candidate peptides scoring process, the communication cost is much less than the computation cost. Eq. (1) indicates that the speedup of CG is nearly CoreNumber when P is negligible because it's not measurable. On the basis of the theoretical analysis and the experimental results in the subsequent sections, we conclude that parallel PSMs algorithm gains better optimization performances with the double buffering mechanism by adequately utilize the advantage of the CG. Table I shows the performance of peptide sequencing with parallel PSMs algorithm before and after the optimization.

## Experimental Results and Discussions

A series of experiments were performed to evaluate the performance and scalability of our proposed SWPepNovo implementation. In this section, we will first introduce the experimental environments and dataset. And then compare the performance of SWPepNovo and some of state-of-the-art de novo peptide sequencing tools. Finally, we evaluate the scalability and accuracy of SWPepNovo.

### Experimental setup

In the experiments, we have implemented and evaluated the parallel PSMs algorithm on Sunway SW26010 many-core architectures. The configuration of the Sunway TaihuLight System listed in the Table II. The experimental spectra data used in the experiments was obtained from *https://www.iprox.org/*, which generated by tandem spectrometry experiment that analyzed a mixture of liver cancer 37. In order to accurately measure the speedup, three different datasets and parameters are used in the experiments, as show as the Table III. We mainly considered the scale of the MS/MS dataset to test the sequencing speed, in which Dataset.1, Dataset.2 and Dataset.3 are acted as small, medium and large computing scale respectively.

### Performance on a single SW26010 node

Firstly, we have compared the single SW26010 performance of proposed SWPepNovo implementation to PepNovo+. Three different datasets (see Table III) were used in the experiments to enhance the accuracy of the experimental results. Note that the entire de novo peptide sequencing progress on a Sunway TaihuLight node with a SW26010 processor.

In order to show the SWPepNovo excelled in speed, we also performed the experiment on PepNovo+, DeepNovo-DIA and Peaks, which executed on an Intel E5-2640 CPU running Linux CentOS 6.5. PepNovo+, operated via command-line interface, is freely available to the researchers. DeepNovo-DIA and PEAKS, the state-of-the-art implementation of de novo peptide sequencing using exhaustive listing of sequences, achieves the optimal performance compared with the existing de novo sequencing methods. Table IV shows the running time comparison between SWPepNovo, PepNovo+, DeepNovo-DIA and PEAKS. As we can see in Table IV, the SWPepNovo spends considerably less than PepNovo+ and Peaks. Notably, in Exp.3, SWPepNovo spent 385 seconds in total, remarkably lower than PepNovo+ 8967 and PEAKS 4521.

**Figure 12:**
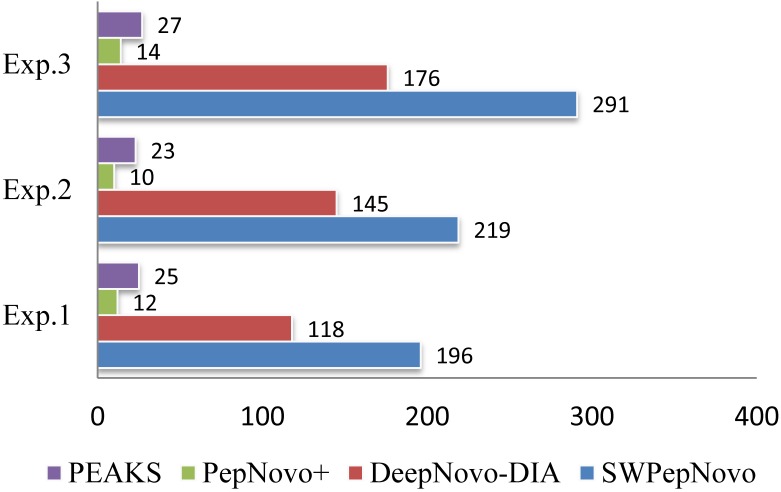
De novo sequencing speeds (spectra/second) of SWPepNovo, PepNovo+ and PEAKS.

Figure 12 illustrates the average speed of SWPepNovo on three different datasets. The average parent precursor mass of the Dataset.3 is 1097 Da, and the length of corresponding average peptide is 10. SWPepNovo can easily de novo peptide sequence more than 291 MS/MS spectrum per second, while PepNovo+ only get in 13 spectrum per second, and PEAKS only get in 25 spectrum per second.

**Figure 13:**
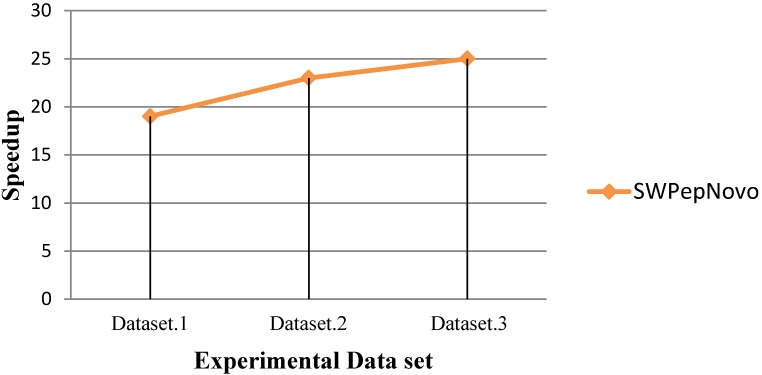
Performance of SWPepNovo.

As Figure 13 shows, SWPepNovo achieves up 28 times speedup on a SW26010 against the PepNovo+. This validates that the parallel PSMs algorithm get high parallel efficiency and speedup ratio using a single SW26010 many-core processor.

#### Performance on the SW26010 cluster

**Figure 14:**
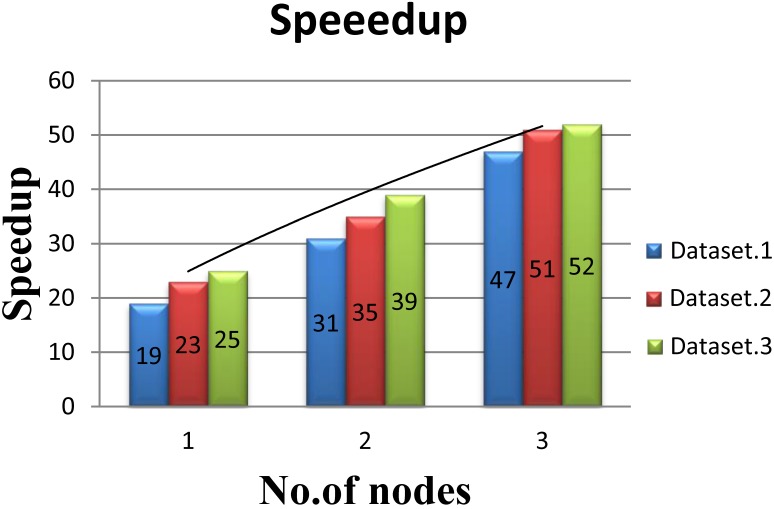
Performance of SWPepNovo on multi-nodes.

In order to evaluate the performance of multi-node acceleration, we have implemented the SWPepNovo on a SW26010 cluster. The impact of the number of nodes in SW26010 cluster on the performance of SWPepNovo is illustrated in Figure 14. As shown in Figure 14, it shows the performance of SWPepNovo against the number of nodes in SW26010 cluster, where the X axis represents the number of SW26010 processor in the cluster and the Y axis represents speedup. In the experiment of three nodes, we got 47 times speedup in Dataset.1, 51 times speedup in Dataset.2 and 52 times in Dataset.3. The performance of SWPepNovo increased with the size of the cluster. The advantage of SWPepNovo over PepNovo+ gets more significant as the cluster size increases.

### Performance for processing large-scale datasets

**Figure 15:**
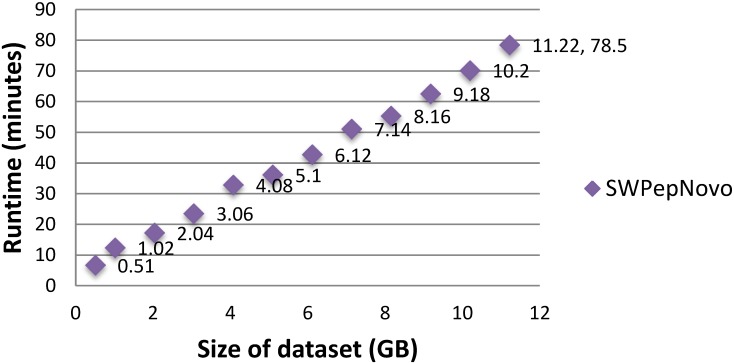
Performance of SWPepNovo on datasets sized 0.51-11.22GB.

To illustrate the large-scale data-processing capacity of our parallel PSMs algorithm, we also performed the experiment on SWPepNovo with extremely large datasets. The extremely large datasets are formed by merging Dataset.1, Dataset.2 and Dataset.3. Figure 15 shows the execution time of SWPepNovo with the dataset size increasing from 0.51GB (120,212 spectra) up to 11.22GB (2,644,664 spectra). With a 11.22 GB of dataset, SWPepNovo took only 78.5 minutes. From Figure 15 we also can see that SWPepNovo+ can de novo sequence extremely large spectra datasets with a linear increase in execution time with the dataset size. Meanwhile, the validity is demonstrated by comparing the SWPepNovo results with PepNovo+. This validates that the parallel PSMs algorithm achieves much higher executive performance than the original serial PSMs algorithm without sacrificing the accuracy and correctness of the de novo peptide sequencing results.

### Accuracy analysis

In this subsection, we verified the accuracy of SWPepNovo by comparing the resultant cosine values of SWPepNovo to PepNovo+. The results are presented in Table V. This validates that SWPepNovo achieves much higher executive performance than PepNovo+ without sacrificing the accuracy and correctness of the results.

## Conclusions

As the size of the MS/MS spectra dataset increases rapidly, the excessive computation time taken by de novo peptide sequencing has become a critical concern in computational biology. This study presents SWPepNovo, a parallel PSMs algorithm to accelerate large-scale de novo peptide sequencing on Sunway TaihuLight Supercomputer. The experimental results demonstrate that the parallel PSMs algorithm can significantly reduce the execution time of large-scale MS/MS spectra analysis without sacrificing accuracy in the results.

## Figures and Tables

**Table A:**
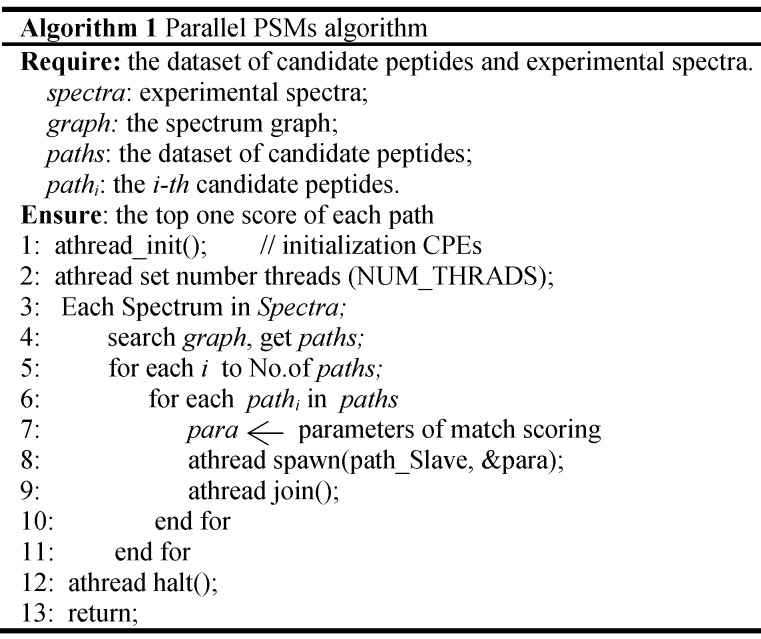
Algorithm 1 Parallel PSMs algorithm

**Table I:** Execution Time Before and After Optimization

Methods	Execution time (seconds)	Benefits
Before Optimization	418s	0
Vectorization	385s	7.8%
Memory Access Optimization	379s	9.3%
Double-buffering	364s	12.9%
With Both Optimization	352s	19.5%

**Table II:** The Sunway TaihuLight System Configuration

CPU	SW26010 processor
Processor Node	4 CGs (4 MPEs and 256 CPEs)
OS	Sunway Raise OS 2.0.5 (based on Linux)
Instruction	Sunway-64 Instruction Set
Compile language	Fortran, C, C++
Parallel programming interface	OpenACC 2.0, OpenMP 3.1, MPI 3.0

**Table III:** De novo peptide sequencing parameters.

Dataset	Instrument	Enzyme	Tolerance	PTMs	Tag length	Size
**Dataset.1**	LTQ	Trypsin	Precursors: 2Da Fragment: 0.75Da	C+57:M+16	6	51.5MB, 18,172 spectra
**Dataset.2**	QSTAR	AspN	Precursors: 2Da Fragment: 0.75Da	C+57:M+16	6	272MB, 52,503 spectra
**Dataset.3**	LTQ	Trypsin	Precursors: 2Da Fragment: 0.75Da	C+57:M+16	6	486MB, 106,616 spectra

**Table IV:** The comparison of running time of three sequencing methods

Methods	Exp.1	Exp.2	Exp.3
PEAKS	728s	2100s	4264s
PepNovo+	1397s	4038s	8188s
DeepNovo-DIA	152s	381s	825 s
SWPepNovo+	73s	175s	352s

**Table V:** Accuracy analysis of SWPepNovo.

Dataset	Cosine of SWPepNovo	Cosine of PepNovo
Dataset 1	0.98546	0.98546
Dataset 2	0.97852	0.97852
Dataset 3	0.98365	0.98365
